# Time-varying model of engagement with digital self reporting: Evidence from smoking cessation longitudinal studies

**DOI:** 10.3389/fdgth.2023.1144081

**Published:** 2023-04-13

**Authors:** Michael Sobolev, Aditi Anand, John J. Dziak, Lindsey N. Potter, Cho Y. Lam, David W. Wetter, Inbal Nahum-Shani

**Affiliations:** ^1^ Cedars-Sinai Medical Center, Los Angeles, CA, United States; ^2^ Institute for Social Research, University of Michigan, Ann Arbor, MI, United States; ^3^ Institute for Health Research and Policy, University of Illinois at Chicago, Chicago, IL, United States; ^4^ Department of Population Health Sciences and Huntsman Cancer Institute, University of Utah, Salt Lake City, UT, United States

**Keywords:** digital intervention, mobile health (mHealth), ecological momentary assessment (EMA), behavior change, habit

## Abstract

**Objective:**

Insufficient engagement is a critical barrier impacting the utility of digital interventions and mobile health assessments. As a result, engagement itself is increasingly becoming a target of studies and interventions. The purpose of this study is to investigate the dynamics of engagement in mobile health data collection by exploring whether, how, and why response to digital self-report prompts change over time in smoking cessation studies.

**Method:**

Data from two ecological momentary assessment (EMA) studies of smoking cessation among diverse smokers attempting to quit (*N* = 573) with a total of 65,974 digital self-report prompts. We operationalize engagement with self-reporting in term of *prompts delivered* and *prompt response* to capture both broad and more granular engagement in self-reporting, respectively. The data were analyzed to describe trends in prompt delivered and prompt response over time. Time-varying effect modeling (TVEM) was employed to investigate the time-varying effects of response to previous prompt and the average response rate on the likelihood of current prompt response.

**Results:**

Although prompt response rates were relatively stable over days in both studies, the proportion of participants with prompts delivered declined steadily over time in one of the studies, indicating that over time, fewer participants charged the device and kept it turned on (necessary to receive at least one prompt per day). Among those who did receive prompts, response rates were relatively stable. In both studies, there is a significant, positive and stable relationship between response to previous prompt and the likelihood of response to current prompt throughout all days of the study. The relationship between the average response rate prior to current prompt and the likelihood of responding to the current prompt was also positive, and increasing with time.

**Conclusion:**

Our study highlights the importance of integrating various indicators to measure engagement in digital self-reporting. Both average response rate and response to previous prompt were highly predictive of response to the next prompt across days in the study. Dynamic patterns of engagement in digital self-reporting can inform the design of new strategies to promote and optimize engagement in digital interventions and mobile health studies.

## 1. Introduction

Digital interventions that leverage mobile and connected devices (e.g., a mobile app to support smoking cessation) have the potential to improve important behavioral and clinical outcomes across a variety of domains. The technology used in digital intervention is increasingly ubiquitous, allowing for granular monitoring of behaviors, experiences and contexts, and facilitating the delivery of real-time interventions in real-world settings (1, 2). Despite this promise, insufficient engagement represents a major barrier that limits the scientific yield from mobile health assessment studies and undermines the effectiveness of digital interventions (1, 3, 4). As a result, there is a growing interest in the “science of engagement” (4–8) in digital health, with research seeking to gain a better understanding of how to conceptualize [e.g., (4)], measure [e.g., (9, 10)] and intervene to promote engagement in mobile health studies and digital interventions [e.g., (11–13)].

We define engagement as a “state of energy investment involving physical, affective, and cognitive energies directed toward a focal stimulus or task” (4). Given challenges in measuring affective and cognitive efforts, mobile health studies mainly focus on the investment of physical energy to measure engagement [e.g., frequency or duration of usage; (8)]. Empirical evidence of objective user engagement suggests that digital interventions and mobile health applications are particularly susceptible to disengagement (3, 14). For example, a large-scale analysis of data from eight remote mobile health studies (15) similarly identified a median retention of only 5.5 days. Engagement with a digital stimulus (e.g., a mobile-based prompt to complete an assessment or to utilize a brief intervention) is particularly challenging in real-world settings due to multiple competing demands on the person's attention, time, and effort, and the ever-increasing number of prompts and notifications from mobile and other digital devices (4). While the general challenge of engagement is well known, the degree to which engagement decreases over time varies significantly between individuals, studies, and mobile apps (3, 15). These challenges highlight the need for empirical work to understand how engagement with a digital stimulus in real-world settings unfolds over time.

Engagement in intensive longitudinal self-reporting *via* digital devices [e.g., *via* ecological momentary assessment (EMAs) or daily diaries] is critical for achieving both research and intervention goals. In terms of research, engagement in intensive longitudinal self-reporting is needed to better understand granular behavior change processes [e.g., how momentary urge to smoke may influence lapse in the next few hours; (16, 17)]. In terms of interventions, engagement is critical for optimizing therapeutic gains from self-monitoring or for leveraging dynamic information about the person's state and context to tailor intervention delivery. Overall, current levels of engagement with digital self-reporting are suboptimal and undermine research and intervention goals. Better understanding of how engagement with a digital stimulus in real-world settings unfolds over time, and what can predict these dynamics patterns, has the potential to inform the development of more dynamic and personalized strategies for promoting engagement in digital interventions and mobile health studies.

Despite the seeming ubiquity of the law of attrition (14) and the engagement challenge in mobile health, substantial variation emerges in time-varying digital self-reporting engagement emerges both between studies and between individuals. An analysis of 8 large-scale mobile health studies with over 100,000 users (15) demonstrated low engagement and rapid attrition while another analysis of 477 studies and 677,536 participants in EMA studies (18) showed high engagement and relatively low attrition. Across the two analyses, the use of monetary incentives emerged as a crucial factor influencing sustained engagement while other study design characteristics had little effect (15, 18). To explain variability between individuals, Pratap et al. (15) identified clusters of individuals based on their tendency to maintain engagement over time. Although this approach can help characterize general patterns of engagement over time, investigating time-varying predictors of engagement can help identify more granular dynamics that likely shape these general patterns.

Focusing on indicators of previous engagement as potential predictors of current engagement can help clarify whether the extent to which previous engagement may undermine or increase future engagement. On the one hand, adhering to the demands of intensive longitudinal data collection may increase burden and hence undermine future engagement (13). On the other hand, individuals are more likely to form a habit to the extent that they adhere to previous self-report prompts which may increase future engagement in self-reporting (4). Given that many studies show decline over time in engagement (15), investigating the association of previous engagement with future engagement throughout the course of a study can shed light on whether and how that association changes over time.

The purpose of this study is to investigate engagement in intensive longitudinal self-reporting over time. Analyzing data from two smoking cessation studies involving EMAs from diverse samples of smokers attempting to quit (*N* = 573), this investigation seeks to uncover granular patterns of engagement that can inform new strategies for promoting engagement. The current paper utilizes two studies with similar procedures, in terms of clinical context and human support, but substantially different populations, duration, and incentive mechanisms for digital self-reporting. As a result, the current paper can provide more generalizable results on engagement over time and highlight conditions under which distinct patterns of engagement emerge. We operationalize engagement with self-reporting in two ways: (a) Prompts delivered: whether or not the participant was prompted *via* the mobile device at least once per day to complete an EMA. Since participants were prompted only if they charged their device and did not turn it off, this operationalization captures broad engagement in self-reporting; and (b) Prompt response: whether or not the participant clicked on the digital prompt, if a prompt was delivered. This operationalization captures more fine-grained engagement in self-reporting among individuals who are relatively engaged (i.e., those for whom prompts were delivered). The current manuscript focuses on addressing two main research questions. First, how does engagement (i.e., prompt delivery and prompt response) with digital self-reporting on cognitions, behaviors, and context change over time as smokers progress through the quit process? Second, do indicators of previous prompt response (i.e., response to the previous prompt, and the history of prompt response prior to current prompt) predict current prompt response, and how do these relationships vary over time as smokers progress through the quit process? Time-varying effect modeling (TVEM) is employed to address these scientific questions.

## 2. Methods

We performed a secondary analysis of data from two smoking cessation studies. These studies enrolled 573 diverse smokers motivated to quit who were asked to self-report their experiences on a digital device and offered nicotine replacement therapy and counseling to help them to quit (Table 1). CARE (R01DA014818, PI: Wetter) included a 5-week digital self-reporting period (1 week pre- and 4 weeks post-cessation). Participants (*N* = 391, mean age = 41, 56% male, 33% African American, 33% non-Hispanic white, 33% Hispanic) were prompted to complete up to 4 digital self-reports randomly delivered throughout the day during normal waking hours. Por Nuestra Salud (PNS; P60MD000503, PI: Wetter) included a 4-week digital self-reporting period (1 week pre- and 3 weeks post-cessation). Participants (*N* = 182, mean age = 42, 62% male, 100% Spanish-speaking Mexican Americans) were prompted to complete up to 3 digital self-reports randomly delivered throughout the day during normal waking hours. More details on the two studies and datasets can be found in Table 1. Previous studies from these data have included models linking socioeconomic status to relapse, as well as longitudinal associations of socioeconomic status, prosmoking social context factors, and affective precipitants with smoking lapse (19–24). The current study is the first to use these datasets to investigate patterns of engagement in digital self-reporting.

**Table 1:** Comparison of CARE and PNS datasets.

	CARE	PNS
Number of participants	391	182
Length of digital self-reporting	1 week pre- and 4 weeks post-cessation	1 week pre- and 3 weeks post-cessation
Number of prompts	4 per day (total number = 56,233)	3 per day (total number = 9,741)
Number of questions per prompt	Between 32 or 43 questions per prompt	44 questions per prompt
Number of prompts per participant during the study	Median = 136; IQR = [84, 188]	Median = 61; IQR = [33, 89]
Response rate	75.83%	68.22%
Population	Diverse population of smokers	Mexican American smokers
Incentive structure for digital self-reporting	Prorated for each week based on random assessments percent completed. Max per week = $50. Max total = $250	Participants compensated $1 per random assessment. Max per week = $21, Max total = $84
Digital self-report technology	Palmtop Personal Computer (PPC)	Palmtop Personal Computer (PPC)
Language	English	Spanish
Demographics	Mean age = 41, 56% male, 33% African American, 33% non-Hispanic white, 33% Hispanic	Mean age = 42, 62% male

### 2.1. Participants

In both the CARE and PNS studies, participants were at least 18 years of age, smoked at least five cigarettes per day over the last year, and were motivated to quit within the next month. Eligible participants were invited to attend an in-person orientation session to provide written informed consent, complete a series of assessments, and receive training in study-related mobile devices and digital self-reporting procedures. All participants received counseling sessions and nicotine patch therapy during the study. All study procedures were approved by the University of Texas MD Anderson Cancer Center Institutional Review Board. Additional details on participants and procedures can be found in previously published papers with the CARE (22) and PNS (24) datasets. For the purpose of this investigation, we restricted the sample to individuals who completed at least one digital self-report during the study period (Table 1).

### 2.2. Digital prompts and self-reports

In the current paper, we analyzed digital prompts that were delivered at random times throughout the day (a total of 65,974 digital self-report prompts across both studies). This design is common in EMA studies and is particularly useful in substance use research which investigates episodic events and relapse (25). In both the CARE and PNS studies, participants were asked to digitally self-report previous smoking events, current urge to smoke, and momentary affect. Both studies also included other types of self-report such as daily dairies and self-initiated reports [see details in (22, 23)].

Both CARE and PNS participants used the same technology for data collection (Table 1). Participants were prompted using a pre-programmed Palmtop Personal Computer (PPC) that they were asked to carry starting 1 week prior to their quit date until the end of the digital self-reporting period. The PPC was a pen-based, touchscreen system that allowed participants to self-initiate or answer survey questions, was extremely user-friendly, and did not require any previous computer or typing skills. The PPC was small (roughly the size of a pack of cigarettes), and participants typically reported no difficulty in carrying it with them at all times. Although PPC is currently an outdated device, it shares many of the capabilities of modern smartphones.

Prompts were delivered only if the participant charged the device and did not turn it off. Each digital self-report prompt was audibly and visually cued by the computer. Audible prompts lasted 30 s. If a participant did not respond to the initial prompt, it was repeated after a 30 s delay. If a participant failed to respond to the second audible prompt, it was repeated again after another 30 s delay. If a participant had still not responded after the third prompt, the digital self-report was recorded as non-response. Because prompts may occur at inconvenient times (e.g., while driving), response to prompts could have been delayed for up to 20 min (4 delays of 5 min each). Any digital self-report not completed during the allotted time was recorded as non-response. The software was designed to ensure that no prompts for digital self-report would occur within 20 min subsequent to any other self-report. Once participants clicked on the prompt, they were presented, one question at a time, between 32 and 44 questions on cognitions, behaviors, and context in both CARE and PNS studies. Participants must either respond or skip a question before the PPC advanced to the next question of the survey. In both studies—CARE and PNS—clicking on the prompt lead to completing the entire assessment questions in 97.16% and 95.46% respectively of digital self-reports. Average time for completion across all days in the study was 3.16 min (SD = 1.55) in CARE and 3.54 min (SD = 1.91) in PNS (Supplementary Materials).

Due to technical problems with the software, the number of delivered prompts per day in the CARE study was higher than the intended 4 during the post-quit period (Supplementary Materials). This deviation may have implications on response rate during the study. We therefore assess the robustness of findings with the PNS dataset which had no observable protocol deviations in terms of the number of prompts.

### 2.3. Compensation

As discussed earlier, CARE and PNS datasets include a diverse sample of smokers and different populations. These datasets were selected also due to the different compensation mechanisms used in each study to incentivize engagement with digital self-reporting. In the CARE study, compensation for digital self-reporting was prorated for each week based on percentage of completed digital self-reports. For example, participants who completed >90% of prompted digital self-report in a particular week received $50 in gift cards for that week while participants who completed 50%–59% of the prompted digital self-reports received $10 in gift cards for that week. In the PNS study, participants were compensated $1 per each completed digital self-report. Additional compensation mechanisms in each study are described in Supplementary Materials.

### 2.4. Operationalizing engagement with digital self-reporting

We operationalize engagement with self-reporting in two ways. The first focused on prompts delivered, indicating whether (= 1) or not (= 0) the participant was prompted *via* the mobile device at least once to complete an EMA on day *d*. This operationalization is motivated by the study designs of CARE and PNS; in both studies, self-reporting prompts were delivered only if the participants charged the device and did not turn it off. The second operationalization focused on prompt response, indicating whether (= 1) or not (= 0) the participant clicked on the digital prompt, if a prompt was delivered at time *t*.

Since the number of prompts delivered would impact the denominator of any analysis that focuses on the rate of prompt response, we first investigated trends in prompts delivered and then investigate trends in prompt response over the course of the two studies. We continue by investigating two predictors of prompt response at time *t*: (a) response to the previous prompt, delivered at time *t *−* *1; and (b) the history of prompt response up to and including time *t *−* *1, computed as the average response rate (i.e., sum of the prompts the participant responded to divided by the total number of prompts delivered up to and including time *t *−* *1).

### 2.5. Analytic plan

We begin by describing trends in prompts delivered over the course of the two studies. We then employ Time-Varying Effect Modeling (TVEM) to investigate trends in prompt response and predictors of prompt response. TVEM is a statistical method that enables health and behavioral scientists to examine dynamic (i.e., time-varying) associations (22, 26–29). TVEM estimates regression coefficients as non-parametric functions of continuous time. First, the overall response rate over the course of the study was modeled as a nonparametric function of time. Second, TVEM was used to estimate the time-specific predictive relationship between response (vs. nonresponse) to the previous prompt, and response (vs. nonresponse) to the current prompt at a given time. This was essentially a marginal logistic regression in which the regression coefficient is allowed to change smoothly but nonlinearly over time. Third, TVEM was used to estimate the relationship between average past response rate (to all previous prompts) and response (vs. nonresponse) to the current prompt. A final TVEM model included both measures as predictors of response vs. nonresponse to the current prompt in order to assess whether considering them both would provide additional predictive information beyond using only one. For each model, within-subject residual correlation was accounted for indirectly by using sandwich (robust) standard errors. TVEM parameter estimates and confidence intervals were plotted to graphically summarize the results. All analyses were completed using the R software and the *tvem* R package (30).

In the current paper, we analyzed a sequence of time-stamped prompts and response outcomes without considering any other time-variant (e.g., affect in previous self-report) or time-invariant covariates (e.g., demographics) in the TVEM models. This allows for our models and findings to be easily reproduced and replicated in any intensive longitudinal or EMA study that employs frequent digital self-reports.

## 3. Results

### 3.1. Prompts delivered

**Figure 1:**
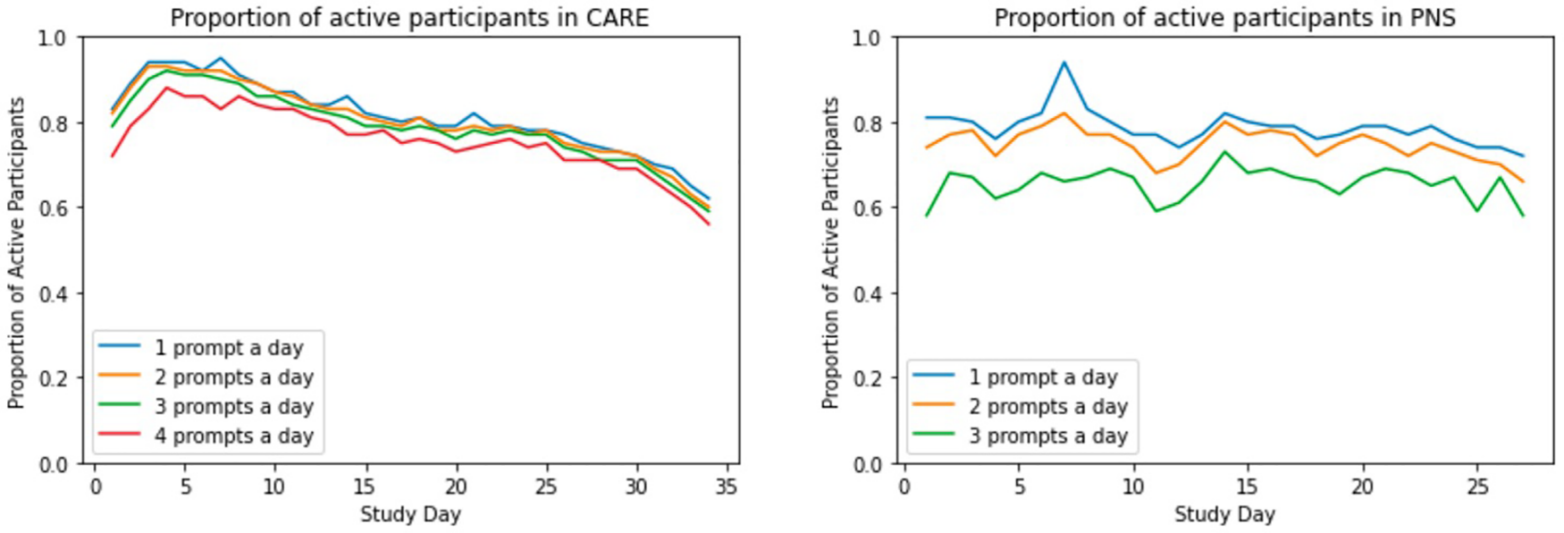
Trends in the proportion of participants with prompts delivered: proportion of participants who received at least 1, 2, 3, or 4 prompts per day in the course of the CARE and PNS studies. Note that in the PNS study the maximum number of prompts per day was 3.

To examine the time-varying role of prompts delivered digital self-reporting in the CARE and PNS studies we computed the percentage of active participants who received at least 1 prompt for each day of the study (i.e., active participants in Figure 1). Because this criterion is relatively lenient, for reference we present in Figure 1 additional analyses for the percentage of active participants who received at least 2, 3, or 4 prompts per day during the CARE and PNS studies. Note that in the PNS study the maximum number of prompts per day was 3. Figure 1 shows the highest proportion of active participants with prompts delivered occurs during the transition from pre-quit to post-quit period (approximately day 7 in the figures below). The proportion of active participants with prompts delivered in PNS is generally lower than in CARE. In CARE, we observe a steady decrease in the proportion of active participants with prompts delivered from approximately day 7 until the end of the study. In PNS, the proportion of active participants with prompts delivered is relatively stable over the course of the study.

### 3.2. Prompt response

The average prompt response across all prompts delivered and days in the CARE study was 75.38% while the average prompt response rate in the PNS dataset was lower at 68.22% (Table 1). These prompt response rates are consistent with aggregate prompt response rates from meta-analyses of EMA and substance use studies (18, 31). We also observe a strong association between response to previous prompts and the likelihood of response to the current prompt. In the CARE dataset, prompt response rate was 85.05% following response to previous prompt, and 47.40% following nonresponse to previous prompt. In the PNS dataset a similar pattern was observed with prompt response rate of 81.92% following response to previous prompt and 38.55% following nonresponse to previous prompt.

**Figure 2:**
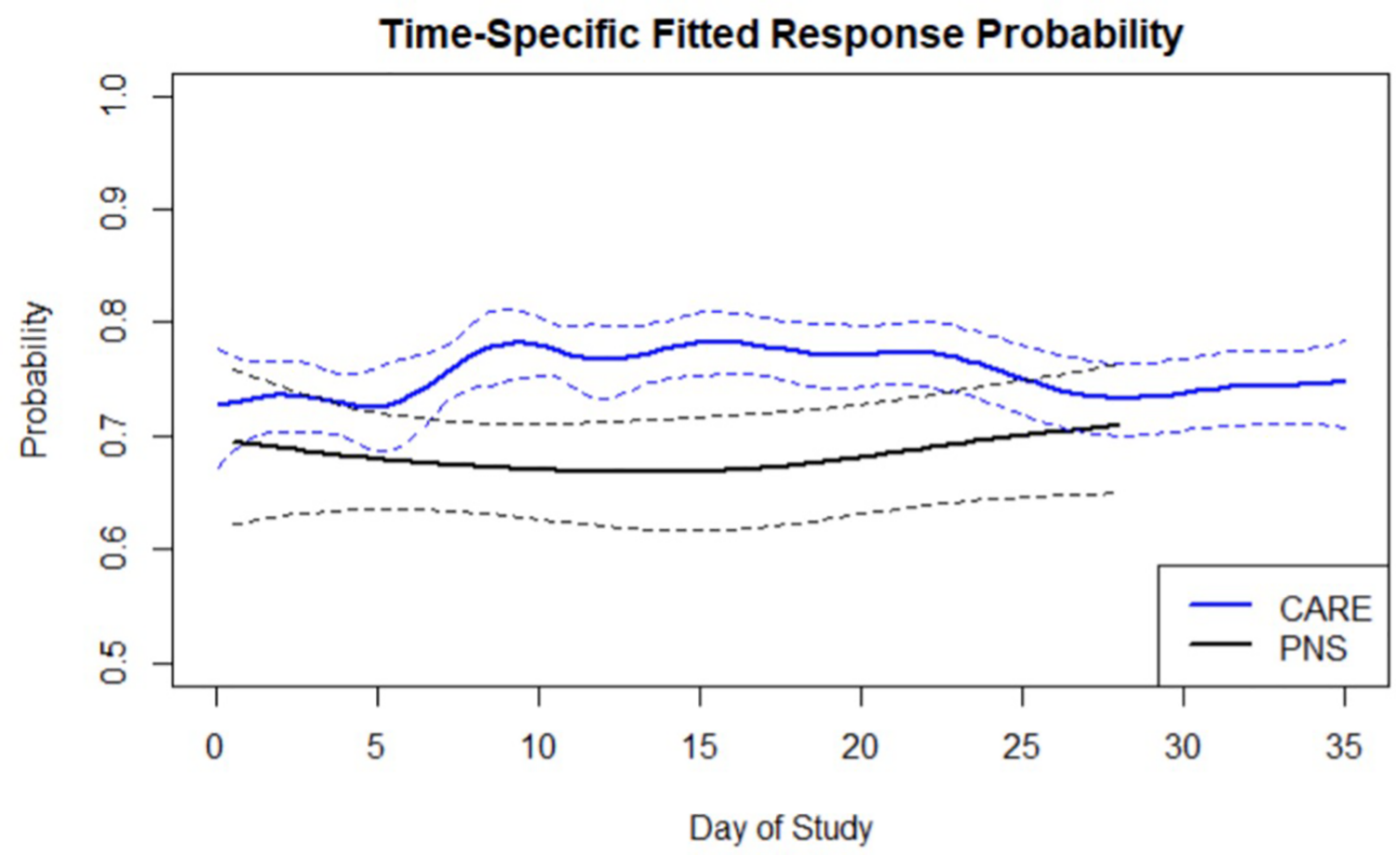
Time-varying response rate across days in the study in the CARE and PNS datasets.

To examine prompt response rate over days in the study, an intercept-only TVEM model was fitted to estimate the average prompt response rate over the study period. Figure 2 displays the estimated average prompt response rate across the study period in both the CARE and PNS data. These smoothed average prompt response probabilities were obtained by using a simple TVEM model with only a time-varying intercept and no other covariate (Model 0). In both the CARE and PNS data, prompt response rate did not appreciably change over the study period and averaged between 65% to 80%. Prompt response rates in the CARE were slightly higher in the first two weeks of post-quit period as opposed to the pre-quit period and the last week of post-quit period. This trend can be attributed to the decline discussed above (Figure 1) in prompts delivered over time. That is, prompt response rate may have remained stable (and slightly increased during the first two weeks of post-quit) because over time only those relatively engaged participants (i.e., those who charged the device and did not turn it off, and thus received at least one prompt per day) remained in the study. Response rates in PNS are significantly lower than response rates in CARE from approximately day 6 to day 21.

#### 3.2.1. Model 1: predicting current prompt response from previous prompt response

**Figure 3:**
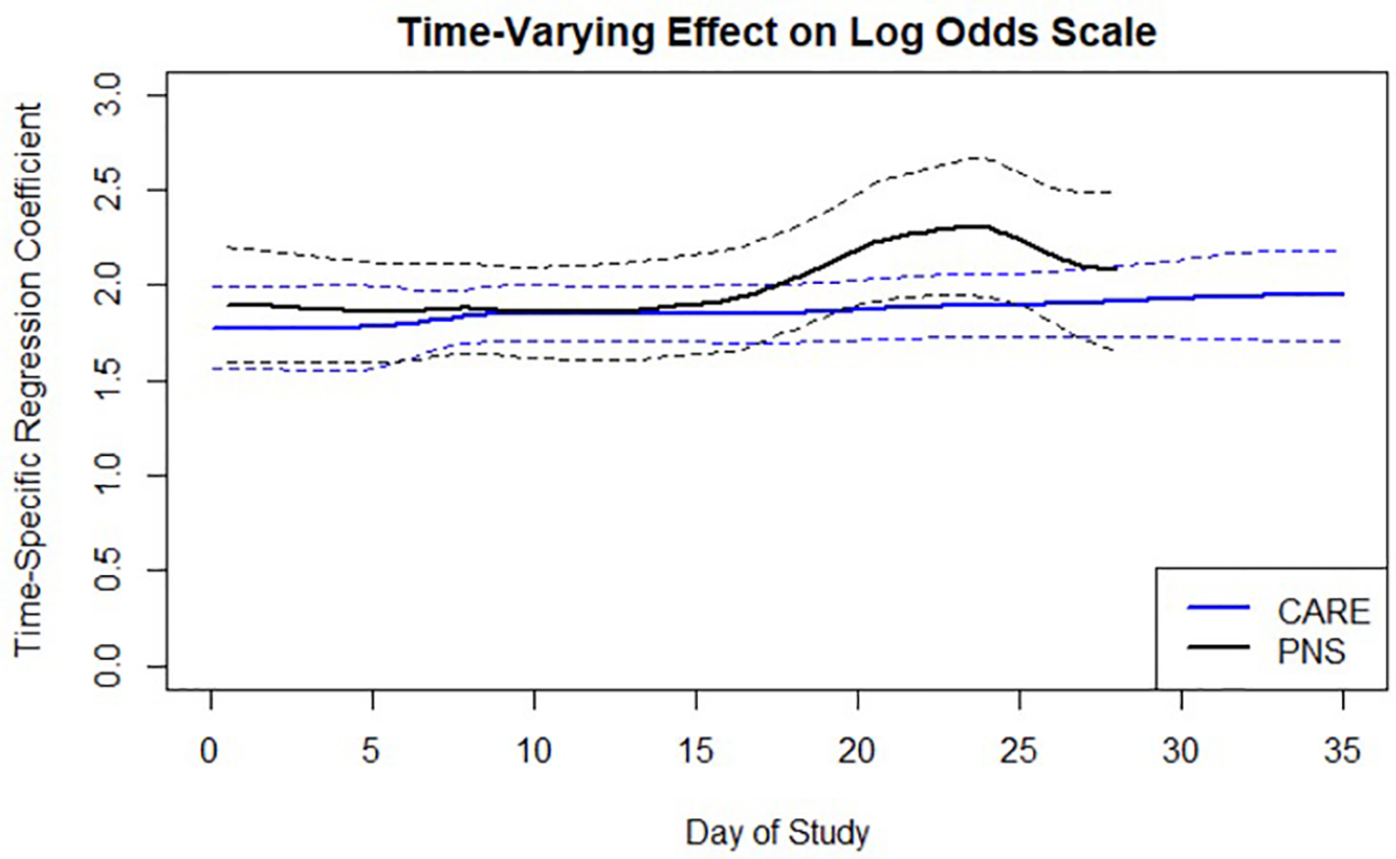
Time-varying relationship between response to previous prompt and likelihood of response to current prompt as function of time in the study.

To examine the effect of previous prompt response, we explored whether response vs. nonresponse to the *j*th prompt by individual *i* at time $t_{ij}$, considered as a binary variable $R_{i}$, is significantly related to response vs. nonresponse to the previous prompt (delivered at time $t_{i,j-1}$), and whether the strength of this relationship changes over the course of the study. In Model 1, a TVEM was fitted to estimate the time-varying association between response to the current prompt, denoted $R_{i}(t_{ij})$, and response to previous prompt, denoted $R_{i}(t_{i,j-1})$, as a function of time $t_{ij}$ in the study. Specifically, it was assumed that

(1) $$\log\mathrm{odds}\;\mathrm{of}\;R_{i}(t_{ij})=\beta_{0}(t_{ij})+\beta_{1}(t_{ij})\times R_{i}(t_{i,j-1})$$

where $R_{i}(t_{ij})$ (current prompt response) and $R_{i}(t_{i,j-1})\;$ (previous prompt response) were intensively measured for subject *i* at many times $t_{ij}$. In Model 1, $\beta_{0}$ is the intercept parameter and represents the log odds of prompt response rate at a given time, supposing that the previous prompt was not responded to [i.e., if $R_{i}(t_{i,j-1})\;$ is set to 0]. $\beta_{1}$ represents the time-varying association between the current response $R_{i}(t_{ij})$ and the previous response $R_{i}(t_{i,j-1})$, and can be interpreted as an estimated time-specific logistic regression coefficient. Models were estimated as a function of days (24 h) since the start of study, resulting in curves of time-varying coefficients $\beta_{0}(t_{ij})$ and $\beta_{1}(t_{ij})$ along a time continuum of 24 h days since the start of the study.$\beta_{1}(t_{ij})$, the relationship between the previous and current prompt response, is plotted in Figure 3. $\beta_{1}(t_{ij})\;$ can be interpreted as the log odds ratio for observing $R_{i}(t_{ij})=1$ given that $R_{i}(t_{i,j-1})\;=\;1$, vs. given that $R_{i}(t_{i,j-1})=0$. The solid and dashed lines represent the fitted coefficients $\beta_{1}(t_{ij})$ and the corresponding pointwise 95% confidence intervals (CIs) respectively. A point on the line represents the time-specific association between current prompt response (at time *t*) and response to the most recent previous prompt. A log odds ratio of significantly greater than 0 indicates a positive predictive relationship, a log odds ratio of significantly less than 0 indicates a negative predictive relationship. The relationship is clearly positive throughout the time interval of the study, and the pointwise CIs never include zero, indicating that the likelihood of a response to the current prompt is higher following a response vs. non-response to the prior prompt. In the CARE dataset, the relationship is roughly stable over time. In the PNS dataset, the relationship is somewhat stronger during days 20–25 than before day 5. The relationship may decrease again after day 25, although this is unclear due to wide CIs.

#### 3.2.2. Model 2: predicting current prompt response from average prompt response rate

**Figure 4:**
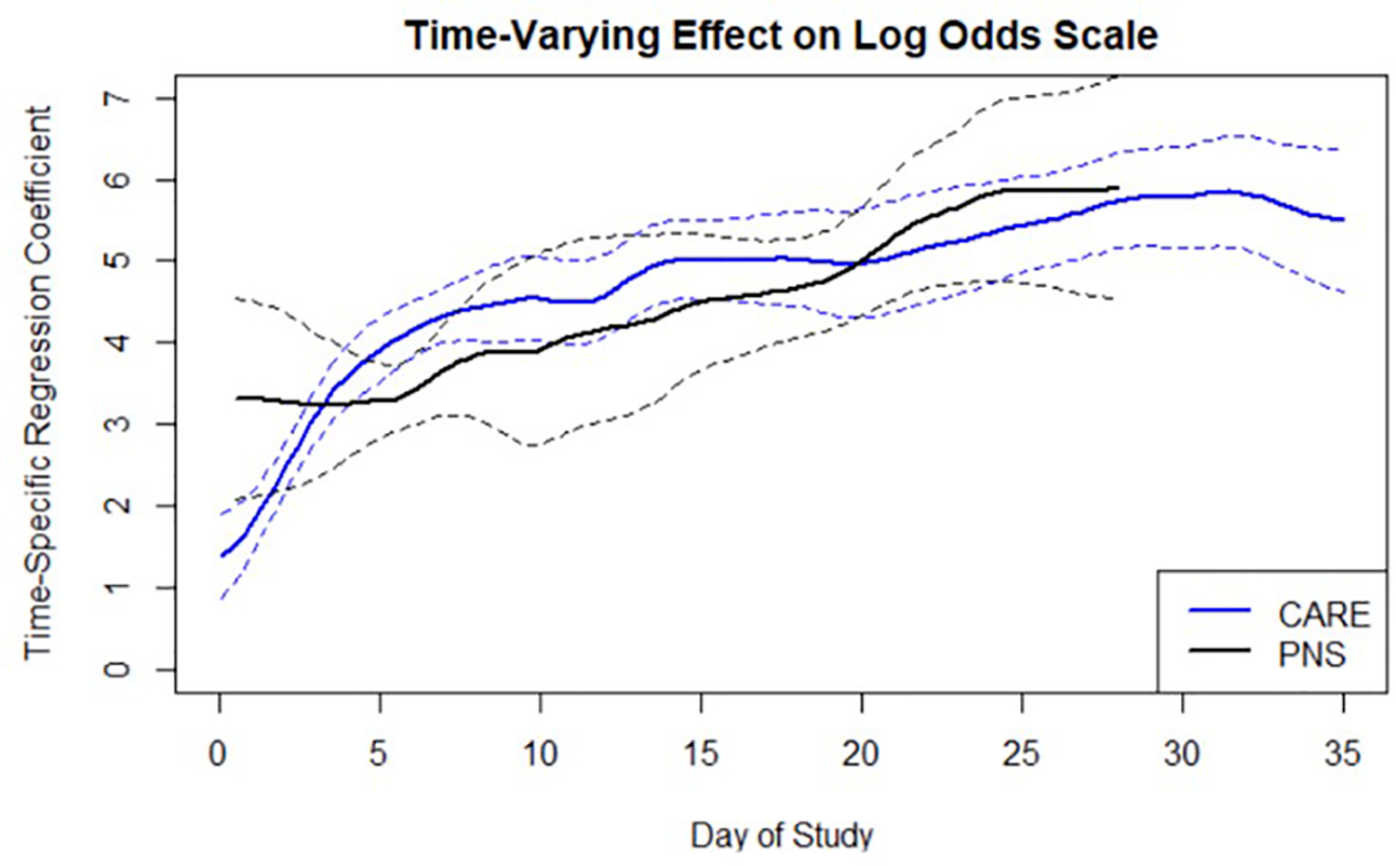
Time-varying relationship between response to current prompt and average prompt response rate prior to current prompt.

To examine the history of prompt response as a potential predictor of current prompt response, we computed the average prompt response rate up to the current prompt and used this variable as a predictor in Model 2:

(2) $$\log\;\mathrm{odds}\;\mathrm{of}\;R_{i}(t_{ij})=\;\beta_{0}(t_{ij})+\beta_{1}(t_{ij})\times H_{i}(t_{i,j-1})\;$$

where $H_{i}(t_{i,j-1})\;$ is the average of all $R_{i}$ observed up to time $t_{i,j-1}$. The coefficient function $\beta_{1}(t_{ij})$ estimated based on Model 2 is shown in the figure below. As plotted in Figure 4, in both CARE and PNS, we observe a positive, significant and increasing relationship between response to current prompt and the average previous prompt response rate, across most of the study period. As expected, this relationship is somewhat weaker in the earliest days of the study, likely because of a shorter history of response or nonresponse to prompts. This observation is generally stronger for CARE compared to PNS. In PNS, the effect of average prompt response rate is initially stronger and less time varying. This could be a result of higher variation in prompt response rates across PNS participants as well as the smaller sample size. The intercept function $\beta_{0}(t_{ij})$ is not shown because it does not have a useful interpretation, since $H_{i}(t_{i,j-1})\;$ is usually not zero.

#### 3.2.3. Model 3: predicting current prompt response from previous prompt response and average response rate

Our final analysis combines Model 1 and Model 2 into a single model to examine the time-varying effect of previous prompt response and prompt response history jointly. That is,

(3) $$\log\mathrm{odds}\;\mathrm{of}\;R_{i}(t_{ij})=\beta_{0}(t_{ij})+\beta_{1}(t_{ij})\times R_{i}(t_{i,j-1})+\beta_{2}(t_{ij})\times H_{i}(t_{i,j-1})\;$$

**Figure 5:**
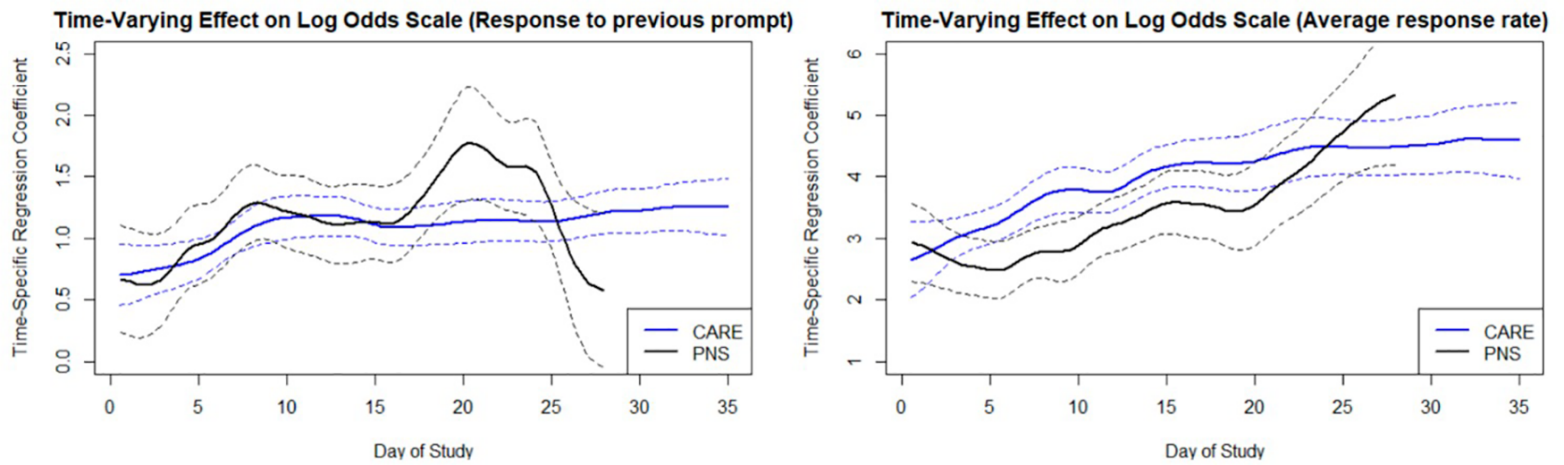
TVEM predicting response to current prompt from response to previous prompt (left) and from the average prompt response rate prior to current prompt (right). The coefficients show the time-varying relationship strength (as time-varying log odds) between response to current prompt and each of the two candidate predictors.

Figure 5 displays the results of Model 3. The figure should be interpreted similarly to the description in Model 1 and Model 2 for the effect of previous prompt response and prompt response history respectively. Overall, the results seem similar between CARE and PNS and consistent with the results described above. The figures below indicates that both previous response and average response rate predict current response across all days in the study, even when controlling for each other.

## 4. Discussion

Research in mobile health and digital interventions highlights the need for empirical work to understand how engagement with a digital stimulus in real-world settings unfolds over time (4, 7). The current paper represents an initial step in addressing a practical and theoretical gap relating to the study of engagement dynamics, by focusing on engagement with digital self-reporting. Consistent with the notion that engagement is a complex construct (4, 32–34), we operationalized engagement in digital self-reporting in two ways. First, given that in the studies of interest participants were prompted only if they charged their device and did not turn it off, we operationalized engagement in terms of whether or not the participant received self-reporting prompts on each specific day during the study (i.e., prompts delivered). Second, we focused on whether or not the participant clicked on the digital prompt when delivered (i.e., prompt response). We discuss the main findings below.

Overall, the results indicate that the proportion of participants with prompts delivered declined steadily over time in the CARE study but not in PNS, but the probability of prompt response was relatively stable across both studies. The lack of decline in prompts delivered in PNS over time can be explained by overall lower initial engagement in PNS compared to CARE in terms of both prompts delivered and prompt response and shorter duration of study. In terms of prompts delivered, at the beginning of the PNS study approximately 60% of the participants received the scheduled number of prompts—this rate is similar to the *lowest* percentage of participants who received the scheduled number of prompts in CARE (i.e., at the last day of the study). In terms of prompt response, the response rate in PNS was lower than CARE in most days of the study. Taken together, this suggests that across both studies the likelihood of clicking on the prompt, if a prompt was delivered, was relatively stable over time. However, the number of prompts delivered was relatively low throughout the study in PNS, and it declined over time in CARE, meaning that fewer participants charged their device and kept it turned on. Below we further discuss engagement patterns and focus on predictors of prompt response engagement.

Our findings also reveal a significant and positive relationship between previous prompt response and current prompt response throughout the data collection period. That is, regardless of time in the study, response (vs. non-response) to a previous prompt was associated with increased likelihood of a response to the current prompt. This pattern of repeated behavior is consistent with a habitual account of engagement with digital stimulus (4). According to basic properties of the psychology of habit (35), behavior is likely to be repeated in a similar context, thereby implying a sequential pattern of response and nonresponse. That is, the relationship between previous and current response is driven by individuals who consistently respond as well as by individuals who consistently do not respond. This relationship can emerge even when average response rates are stable over time. In the current analysis, we find no consistent increasing effect in the first days of the study which suggests a very quick formation of response or non-response habit.

Our findings also reveal a strong, positive, and increasing relationship between the history of prompt response (i.e., the average response rate to prior prompts) and likelihood of responding to the next prompt throughout the time interval of the study. We observed an increasing pattern that does not seem to plateau even after more than 100 delivered prompts which further highlight the importance of this feature in time-varying prediction of engagement. To investigate the generalizability of this finding, future research should examine the extent that the frequency of assessment and length of data collection impact the speed of habit formation and shape of the curve over time (e.g., when does the effect of average response rate plateau).

Our findings also contribute to the debate concerning whether engagement is a temporally dynamic state, a relatively stable trait, or both (4). Our analysis suggests that engagement can be predicted by indicators of state (e.g., previous response) and trait (e.g., average response rate), even when controlling for each other in the same model. These insights can help develop interventions to promote engagement. Specifically, understanding time-varying indicators of engagement has the potential to inform the development of more dynamic and personalized strategies for promoting engagement. Our results highlight two potential time-varying tailoring variables (i.e., response to previous prompt and average response rate) that can be further studied using micro-randomized trials [MRTs: (36)] to optimize the delivery of just-in-time adaptive interventions (4) for promoting engagement and preventing disengagement. For example, an MRT could be designed to investigate whether and what type of an intervention should be triggered when an individual does not respond to a given prompt and/or when their response rate falls below a specific threshold [see other examples: (11, 13, 37)]. Using mobile health technology, such MRT could evaluate a variety of novel real-time engagement strategies [e.g., delivering summaries of self-reported data; (13)] beyond the incentives used in the current study. We discuss additional engagement strategies and use of incentives below.

These results highlight the importance of systematically integrating multiple indicators to measure engagement in self-reporting. For example, a latent construct that captures both the performance of activities that are required for the prompt to be sent (e.g., charging the device, turning the device on) as well as whether or not the person responded to the prompt if a prompt was sent, will likely provide a more valid measure of engagement in self-reporting. Similarity, integrating indicators of cognitive (e.g., variance in response to questions) and affective (e.g., appreciation of the benefits associated with survey completion) energy investment will likely provide a more valid measure of engagement in self-reporting (4), compared to focusing on the investment of physical energy alone (e.g., responding to the prompt). The integration of multiple indicators will likely yield additional and more generalizable insights about the dynamics of engagement in self-reporting. Collecting qualitative data from participants on reasons for disengaging can further support a more comprehensive analysis of engagement patterns. Future research should focus on developing more comprehensive ways to measure and model engagement in self-reporting.

Beyond advancements in measurement of engagement it is also crucial to design and evaluate strategies that can increase engagement with the digital device used in the study. For example, providing human support can increase accountability, and delivering summaries and visualization of previously collected data can increase the value of engaging with the study device and thus improve engagement (13). In term of study design, investigators should strive to identify the ideal number of prompts per day and questions per prompt that will maximize scientific yield while minimizing participant burden (38). Future work should examine the effectiveness of these strategies and others [see (4)] in promoting engagement in mobile health data collection studies.

One of the best practices in mobile health data collection is the use of contingent monetary incentives for completing digital self-report (39). According to two recent meta-analyses (18, 31), response rates in studies that include contingent monetary incentives range around 75%. Albeit relatively high, these rates can be considered suboptimal given the high financial incentives typically provided to encourage response (e.g., up to $250 total for 36 days in CARE). Response rates tend to be lower when minimal financial incentives are provided, especially when the number of assessments is large (18). However, the use of monetary incentives to enhance engagement in intensive longitudinal self-reporting may backfire as it increases the overall cost of the study (or the intervention) and hence undermines the feasibility of future replications (or the scalability of the intervention). High monetary incentives may also undermine intrinsic motivation which can hinder completion rates in future studies (40). There is a need to design mobile health studies that identify and address insufficient engagement while minimizing the use of monetary incentives for completing digital self-reports (13, 37).

Beyond operationalization of engagement that we discuss above, the current paper has several additional limitations. First, our findings on time-varying prompt response patterns should be interpreted cautiously because TVEM cannot distinguish between-subject and within-subject effects. In particular, there are at least three explanations for why responding to a previous prompt predicts responding to the current prompt. Response to the previous prompt could be either a within-person confounder (because both prompts are delivered in a similar personal state and context) or a between-person confounder (because there might be diverging profiles of people who are more or less engaged), or else there could be a direct causal relationship from previous to current response. These explanations cannot be clearly distinguished in observational data and would require design of experiments to disentangle these potential explanations. Second, as discussed in the method section, bugs in the data collection software impacted the data collection protocol in one of the studies. Specifically, in CARE, the number of actual prompts triggered during the post-quit period was significantly higher than intended by the study design. This deviation from the protocol had the potential to increase participant burden and thus undermine engagement. However, results from the CARE study indicated stable prompt response over time, even during the period of higher number of prompts. Our main findings are also replicated in the PNS study which did not have any detected deviation from the protocol. A final limitation include generalization to engagement with digital self-reporting with smartphones and mobile health apps. In our study, as in many other mobile health studies, participants received the study device and could disengage from digital self-reporting by turning the device off or not charging it. This type of disengagement is presumably less likely when participants are using their own mobile devices for digital self-reporting. In fact, recent research demonstrated greater engagement with “bring-your-own device” clinical research as opposed to study-provided devices. Bring-your-own-device method potentially increase engagement because they tend to be more user-friendly and allow participants to use technologies that they are familiar with (41, 42). Future research should examine whether our results generalize to this setting as well.

In conclusion, the current paper highlighted the importance of integrating various indicators to measure engagement in digital self-reporting. The results indicate that although engagement in terms of prompt response remained stable over time, engagement in terms of prompts delivered declines steadily. Thus, focusing on one indicator alone (e.g., prompt response) may lead to misleading conclusions about trends in engagement with self-reporting. Both the person's response to previous prompt and prompt response history were found as salient predictors of response to a given prompt. The results can be used to guide the design of future studies to optimize the delivery of real-time interventions for promoting engagement, thereby enhancing the utility of digital interventions and mobile health studies.

## Data Availability

Deidentified, limited datasets are available upon request.

## References

[1] Michie S Yardley L West R Patrick K Greaves F. Developing and evaluating digital interventions to promote behavior change in health and health care: recommendations resulting from an international workshop. J Med Internet Res. (2017) 19(6):e232. 10.2196/jmir.7126 28663162 PMC5509948

[2] Nahum-Shani I Smith SN Spring BJ Collins LM Witkiewitz K Tewari A Just-in-time adaptive interventions (JITAIs) in mobile health: key components and design principles for ongoing health behavior support. Ann Behav Med. (2018) 52(6):446–62. 10.1007/s12160-016-9830-8 27663578 PMC5364076

[3] Baumel A Muench F Edan S Kane JM. Objective user engagement with mental health apps: systematic search and panel-based usage analysis. J Med Internet Res. (2019) 21(9):e14567. 10.2196/14567 31573916 PMC6785720

[4] Nahum-Shani I Shaw SD Carpenter SM Murphy SA Yoon C. Engagement in digital interventions. Am Psychol. (2022) 77(7):836. 10.1037/amp0000983 35298199 PMC9481750

[5] Rabbi M Li K Yan HY Hall K Klasnja P Murphy S. Revibe: a context-assisted evening recall approach to improve self-report adherence. Proc ACM Interact Mob Wearable Ubiquitous Technol. (2019) 3(4):1–27. 10.1145/3369806 34164595 PMC8218636

[6] Yeager CM Benight CC. If we build it, will they come? Issues of engagement with digital health interventions for trauma recovery. MHealth. (2018) 4:37. 10.21037/mhealth.2018.08.04 30363749 PMC6182033

[7] Yeager CM Benight CC. Engagement, predictors, and outcomes of a trauma recovery digital mental health intervention: longitudinal study. JMIR Ment Health. (2022) 9(5):e35048. 10.2196/35048 35499857 PMC9112079

[8] Rabbi M Kotov MP Cunningham R Bonar EE Nahum-Shani I Klasnja P Toward increasing engagement in substance use data collection: development of the Substance Abuse Research Assistant app and protocol for a microrandomized trial using adolescents and emerging adults. JMIR research protocols. (2018) 7(7):e9850. 10.2196/resprot.9850 PMC6070723 30021714

[9] Perski O Blandford A West R Michie S. Conceptualising engagement with digital behaviour change interventions: a systematic review using principles from critical interpretive synthesis. Transl Behav Med. (2017) 7(2):254–67. 10.1007/s13142-016-0453-1 27966189 PMC5526809

[10] Perski O Blandford A Garnett C Crane D West R Michie S. A self-report measure of engagement with digital behavior change interventions (DBCIs): development and psychometric evaluation of the “DBCI engagement scale”. Transl Behav Med. (2019) 10(1):267–77. 10.1093/tbm/ibz039 PMC8411853 30927357

[11] Bidargaddi N Almirall D Murphy S Nahum-Shani I Kovalcik M Pituch T To prompt or not to prompt? A microrandomized trial of time-varying push notifications to increase proximal engagement with a mobile health app. JMIR Mhealth Uhealth. (2018) 6(11):e10123. 10.2196/10123 30497999 PMC6293241

[12] Militello L Sobolev M Okeke F Adler DA Nahum-Shani I. Digital prompts to increase engagement with the headspace app and for stress regulation among parents: feasibility study. JMIR Form Res. (2022) 6(3):e30606. 10.2196/30606 35311675 PMC8981020

[13] Nahum-Shani I Rabbi M Yap J Philyaw-Kotov ML Klasnja P Bonar EE Translating strategies for promoting engagement in mobile health: a proof-of-concept microrandomized trial. Health Psychol. (2021) 40(12):974–87. 10.1037/hea0001101 34735165 PMC8738098

[14] Eysenbach G. The law of attrition. J Med Internet Res. (2005) 7(1):e11. 10.2196/jmir.7.1.e11 15829473 PMC1550631

[15] Pratap A Neto EC Snyder P Stepnowsky C Elhadad N Grant D Indicators of retention in remote digital health studies: a cross-study evaluation of 100,000 participants. NPJ Digit Med. (2020) 3(1):1–10. 10.1038/s41746-020-0224-8 32128451 PMC7026051

[16] Joseph NT Jiang Y Zilioli S. Momentary emotions and salivary cortisol: a systematic review and meta-analysis of ecological momentary assessment studies. Neurosci Biobehav Rev. (2021) 125:365–79. 10.1016/j.neubiorev.2021.02.042 33662445

[17] Shiffman S Stone AA Hufford MR. Ecological momentary assessment. Annu Rev Clin Psychol. (2008) 4:1–32. 10.1146/annurev.clinpsy.3.022806.091415 18509902

[18] Wrzus C Neubauer AB. Ecological momentary assessment: a meta-analysis on designs, samples, and compliance across research fields. Assessment. (2022) 30(3):825–46. 10.1177/10731911211067538 35016567 PMC9999286

[19] Cambron C Haslam AK Baucom BRW Lam C Vinci C Cinciripini P Momentary precipitants connecting stress and smoking lapse during a quit attempt. Health Psychol. (2019) 38(12):1049–58. 10.1037/hea0000797 31556660 PMC6861642

[20] Cambron C Lam CY Cinciripini P Li L Wetter DW. Socioeconomic status, social context, and smoking lapse during a quit attempt: an ecological momentary assessment study. Ann Behav Med. (2019) 54(3):141–50. 10.1093/abm/kaz034 PMC7455803 31612218

[21] Hopkins PD Spears CA Hoover DS Li L Cambron C Potter LN Trajectories of motivation and self-efficacy during a smoking quit attempt: an ecological momentary assessment study. Psychol Addict Behav. (2022) 36(1):78–89. 10.1037/adb0000734 34435832 PMC11495658

[22] Potter LN Haaland BA Lam CY Cambron C Schlechter CR Cinciripini PM A time-varying model of the dynamics of smoking lapse. Health Psychol. (2021) 40(1):40–50. 10.1037/hea0001036 33370151 PMC8265776

[23] Vinci C Li L Wu C Lam CY Guo L Correa-Fernández V The association of positive emotion and first smoking lapse: an ecological momentary assessment study. Health Psychol. (2017) 36(11):1038–46. 10.1037/hea0000535 28726478 PMC5653435

[24] Vinci C Guo L Spears CA Li L Correa-Fernández V Etcheverry PE Socioeconomic indicators as predictors of smoking cessation among spanish-speaking Mexican Americans. Ethn Health. (2019) 24(7):841–53. 10.1080/13557858.2017.1373074 28859518 PMC5832556

[25] Shiffman S. Ecological momentary assessment (EMA) in studies of substance use. Psychol Assess. (2009) 21(4):486–97. 10.1037/a0017074 19947783 PMC2846437

[26] Lanza ST Vasilenko SA Liu X Li R Piper ME. Advancing the understanding of craving during smoking cessation attempts: a demonstration of the time-varying effect model. Nicotine Tob Res. (2014) 16(Suppl 2):S127–134. 10.1093/ntr/ntt128 23975881 PMC3977629

[27] Lanza ST Vasilenko SA Russell MA. Time-varying effect modeling to address new questions in behavioral research: examples in marijuana use. Psychol Addict Behav. (2016) 30(8):939–54. 10.1037/adb0000208 27736149 PMC5222706

[28] Shiyko MP Lanza ST Tan X Li R Shiffman S. Using the time-varying effect model (TVEM) to examine dynamic associations between negative affect and self confidence on smoking urges: differences between successful quitters and relapsers. Prev Sci. (2012) 13(3):288–99. 10.1007/s11121-011-0264-z 22246429 PMC3372905

[29] Tan X Shiyko MP Li R Li Y Dierker L. A time-varying effect model for intensive longitudinal data. Psychol Methods. (2012) 17(1):61–77. 10.1037/a0025814 22103434 PMC3288551

[30] Dziak JJ Coffman DL Li R Litson K Chakraborti Y. *tvem: Time-Varying Effect Models* (1.3.1) (2021). Available at: https://CRAN.R-project.org/package=tvem 10.1080/00273171.2022.2149449 PMC10966971 36622859

[31] Jones A Remmerswaal D Verveer I Robinson E Franken IHA Wen CKF Compliance with ecological momentary assessment protocols in substance users: a meta-analysis. Addiction. (2019) 114(4):609–19. 10.1111/add.14503 30461120 PMC6492133

[32] Bijkerk LE Oenema A Geschwind N Spigt M. Measuring engagement with mental health and behavior change interventions: an integrative review of methods and instruments. Int J Behav Med. (2022) 30(2):155–66. 10.1007/s12529-022-10086-6 35578099 PMC10036274

[33] Kniestedt I Lefter I Lukosch S Brazier FM. Re-framing engagement for applied games: a conceptual framework. Entertain Comput. (2022) 41:100475. 10.1016/j.entcom.2021.100475

[34] Oertel C Castellano G Chetouani M Nasir J Obaid M Pelachaud C Engagement in human-agent interaction: an overview. Front Robot AI. (2020) 7:92. 10.3389/frobt.2020.00092 33501259 PMC7806067

[35] Wood W Rünger D. Psychology of habit. Annu Rev Psychol. (2016) 67:289–314. 10.1146/annurev-psych-122414-033417 26361052

[36] Qian T Walton AE Collins LM Klasnja P Lanza ST Nahum-Shani I The microrandomized trial for developing digital interventions: experimental design and data analysis considerations. Psychol Methods. (2022) 27(5):874–94. 10.1037/met0000283 35025583 PMC9276848

[37] Carpenter SM Yap J Patrick ME Morrell N Dziak JJ Almirall D Self-relevant appeals to engage in self-monitoring of alcohol use: a microrandomized trial. Psychol Addict Behav. (2022). 10.1037/adb0000855 35834200 PMC9843482

[38] Schneider S Junghaenel DU Smyth JM Fred Wen CK Stone AA. Just-in-time adaptive ecological momentary assessment (JITA-EMA). Behav Res Methods. (2023). 10.3758/s13428-023-02083-8 36840916 PMC10450096

[39] Burke LE Shiffman S Music E Styn MA Kriska A Smailagic A Ecological momentary assessment in behavioral research: addressing technological and human participant challenges. J Med Internet Res. (2017) 19(3):e7138. 10.2196/jmir.7138 PMC5371716 28298264

[40] Gneezy U Meier S Rey-Biel P. When and why incentives (don’t) work to modify behavior. J Econ Perspect. (2011) 25(4):191–210. 10.1257/jep.25.4.191

[41] Demanuele C Lokker C Jhaveri K Georgiev P Sezgin E Geoghegan C Considerations for conducting bring your own “device” (BYOD) clinical studies. Digit Biomark. (2022) 6(2):47–60. 10.1159/000525080 35949223 PMC9294934

[42] Pugliese L Woodriff M Crowley O Lam V Sohn J Bradley S. Feasibility of the “bring your own device” model in clinical research: results from a randomized controlled pilot study of a mobile patient engagement tool. Cureus. (2016) 8(3):e535. 10.7759/cureus.535 27096135 PMC4835151

