# Supplementary material for: Time-varying model of engagement with digital self reporting: Evidence from smoking cessation longitudinal studies
Source: Front Digit Health. 2023 Apr 13;5:1144081. doi: 10.3389/fdgth.2023.1144081 (PMC10134394; doi:10.3389/fdgth.2023.1144081)
Supplement: Supplementary file 1 [file Datasheet1.pdf]

Supplementary Material

Supplementary Material for Sobolev et al. (2023) “Time-Varying Model of Engagement with Digital Self Reporting: Evidence from Smoking Cessation Longitudinal Studies”

1 Compensation

In both CARE and PNS studies, participants received financial compensation at each clinic visit to compensate for the time and inconvenience associated with their participation in the study such as transportation, child-care, etc. Compensation was provided for data collection only and was not associated with treatment. Compensation was provided in the form of gift cards to Target and/or Wal-Mart.

1.1 CARE

Participants were reimbursed $20 in gift cards for each clinic visit through week 2; week four and 26 visits were reimbursed at $40 in gift cards each. Participants who attend all six study visits received $180 in gift cards (5 study visits x $20; 2 study visits at $40).

Participants were eligible for additional compensation of up to $50 in gift cards per week for their time completing the EMA procedures. Compensation for the EMA procedures were prorated for each week based on percent completed random assessments. Participants who completed >90% of the random assessments in a particular week received $50 in gift cards for that week. Participants who completed 75-89% of the random assessments received $35 in gift cards for that week. Participants who completed 60-74% of the random assessments received $20 in gift cards for that week. Participants who completed 50-59% of the random assessments received $10 in gift cards for that week. Because participants carried the PPC for 5 weeks (1 weeks precessation through 4 weeks postcessation), they were eligible to receive $250 in gift cards for the EMA procedures. Thus, participants could have received a total of $430 in gift cards for completing all the study procedures including the clinic visits and EMA procedures.

1.2 PNS

Participants were be reimbursed $20 for the first four in-person visits (Orientation, Baseline, Week 0, and Week 3), and $50 for the final in-person follow-up visit (Week 26). Thus, participants in the main study who completed all study visits received a total of $130.

Participants were eligible for additional compensation of up to $35 per week for their time completing the EMA procedures. Participants were compensated $1 per random assessment and daily diary completed. There were 3 random assessments per day and 1 diary per day for a total of 4 weeks. Thus, participants were compensated up to $140 for the EMA procedures ($5 per day X 7 days/week X 4 weeks) and up to a total of $270 for completion of all study procedures including the clinic visits, telephone assessment, and EMA procedures.

2 Additional Descriptive Statistics for CARE and PNS

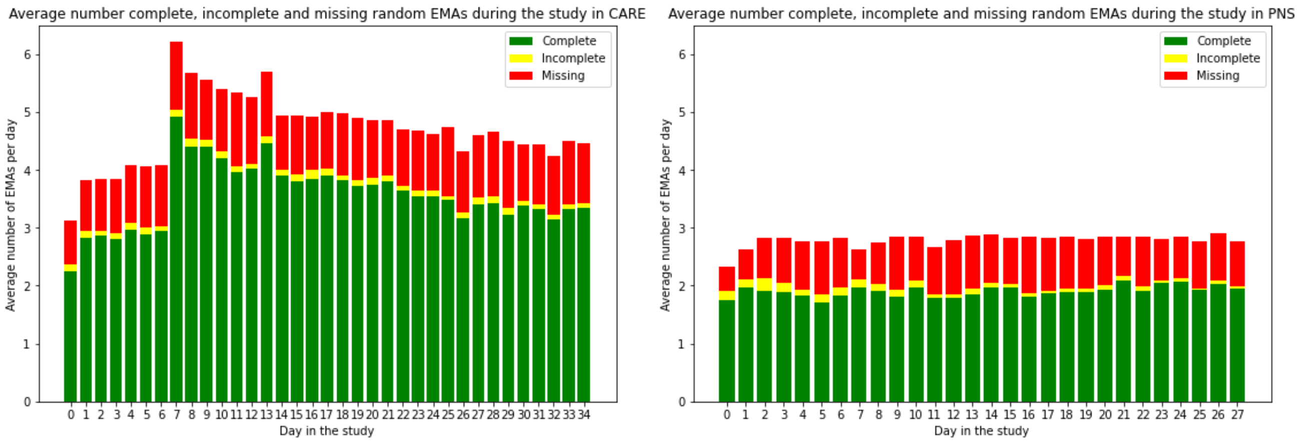

Supplementary Figure 1. Average number of complete, incomplete and missing digital self-reports during the CARE and PNS studies.

The intercept-only TVEM below shows the estimated average completion time for digital self-reporting assessment over time in the study. In the CARE dataset, it indicates average completion time of around 4 minutes in the beginning of the study and convergence to around 3 minutes after 5 days. In the PNS dataset, it indicates average completion time of around 5 minutes in the beginning of the study and a slower convergence to around 3 minutes after 15 days.

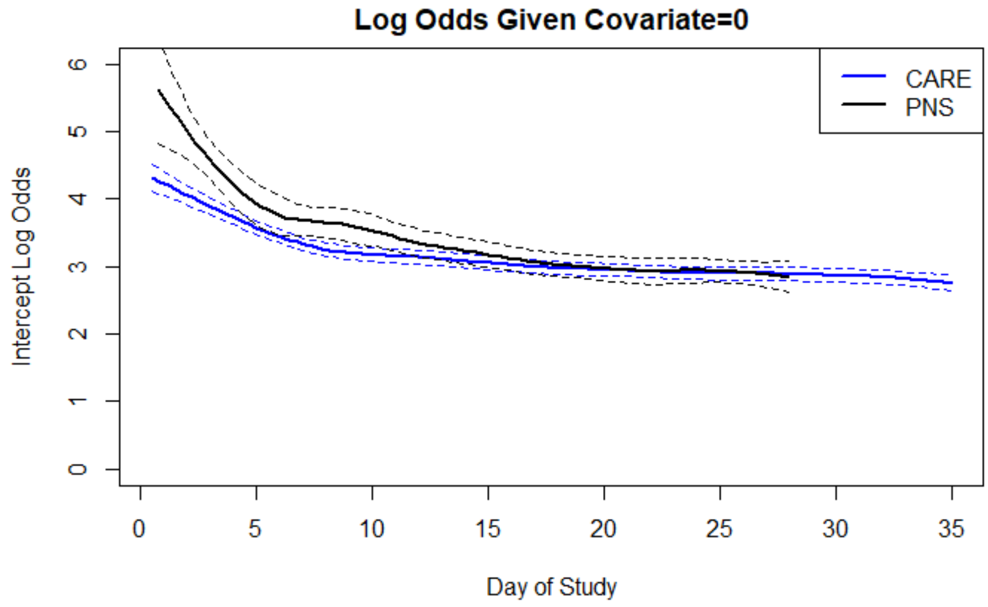

Supplementary Figure 2. Average time (in minutes) to complete the digital self-reporting assessment in the CARE and PNS datasets.

3 Sensitivity Analysis for Active Users

A sensitivity analysis was performed to examine whether the results of the models remain approximately the same when filtering for users who were active (i.e., had at least one prompt each day of the study) during the entire duration of each study. Filtering the results based on this criterion resulted in a N=142 for CARE and only N=42 for PNS. The TVEM models results are presented below.

3.1 Model 0

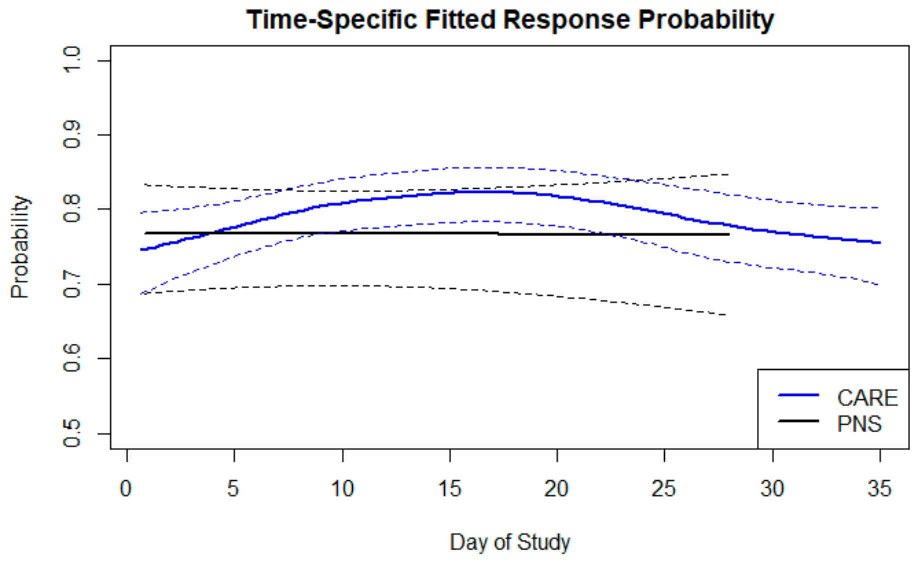

Supplementary Figure 3. Time-varying response rate across days in the study in the CARE and PNS datasets.

3.2 Model 1

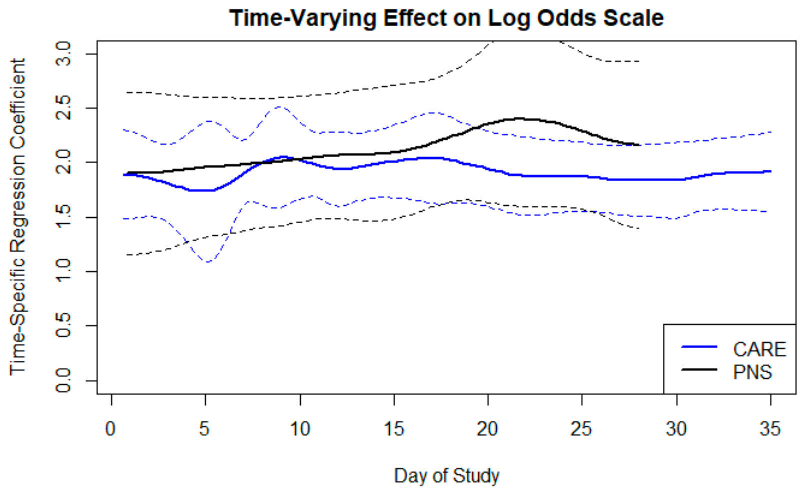

Supplementary Figure 4. Time-varying relationship between response to previous prompt and likelihood of response to current prompt ($R_i(t_{ij})$) as function of time t_{ij} in the study.

3.3 Model 2

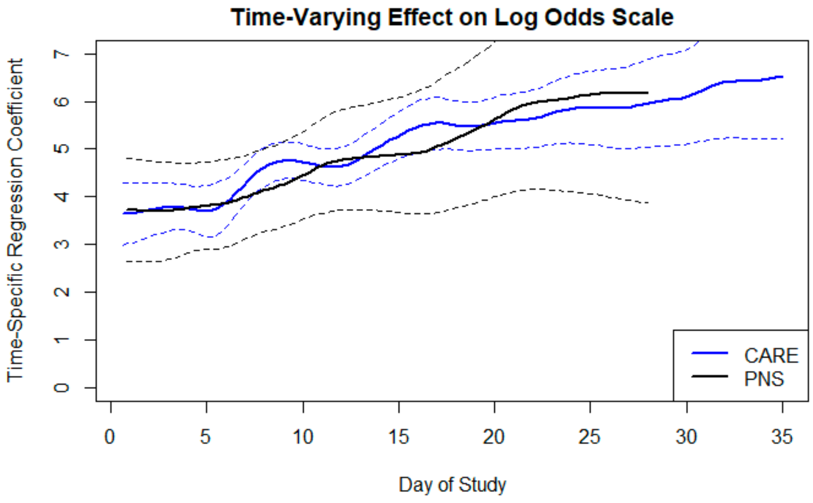

Supplementary Figure 5. Time-varying relationship between response to current prompt (t) and average response rate until time t.

3.4 Model 3

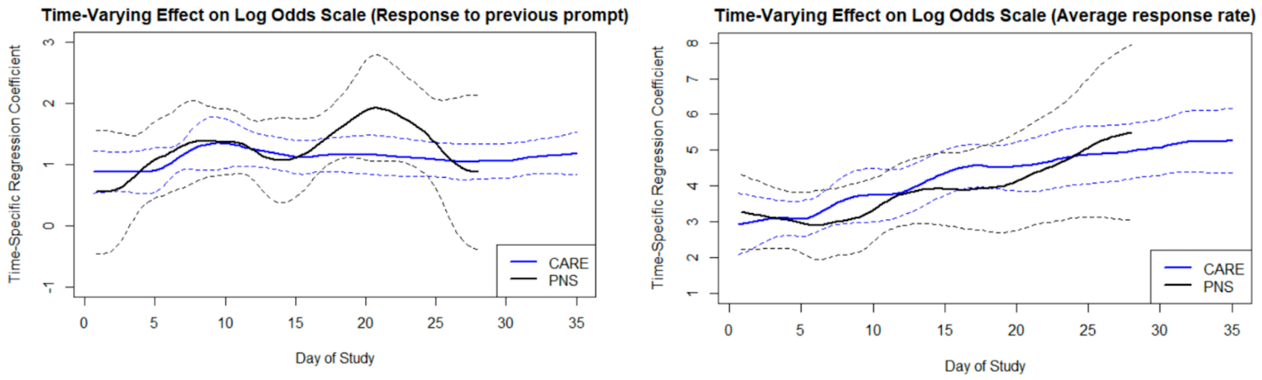

Supplementary Figure 6. TVEM predicting response to current prompt (at time t) from response to previous prompt $R_i(t_{i,j-1})$ and from average response rate $H_{ij}(t_{ij})$ before time t. The coefficients show the time-varying relationship strength (as time-varying log odds) between response to current prompt and each of the two predictor variables.

4 Temporal Dynamics

The time axis in the previous models represented time (in days) since the start of the study. Another way in which time might affect the relationship between previous and current EMA, can be viewed in terms of the random EMA prompts, which might be closer or further away in time. The time since the previous EMA was triggered (0-24 hours) was used in the TVEM models for the following figures.

The intercept-only TVEM below shows the estimated probability as a function of time since the previous prompt, marginally (i.e., on average) over overall time in the study. In the CARE dataset, it indicates that a response is especially likely when the time difference between the two prompts is less than a couple of hours and response is less likely when the difference is between 5 to 10 hours. In the PNS dataset, response rate does not change significantly as a function of time since the previous prompt.

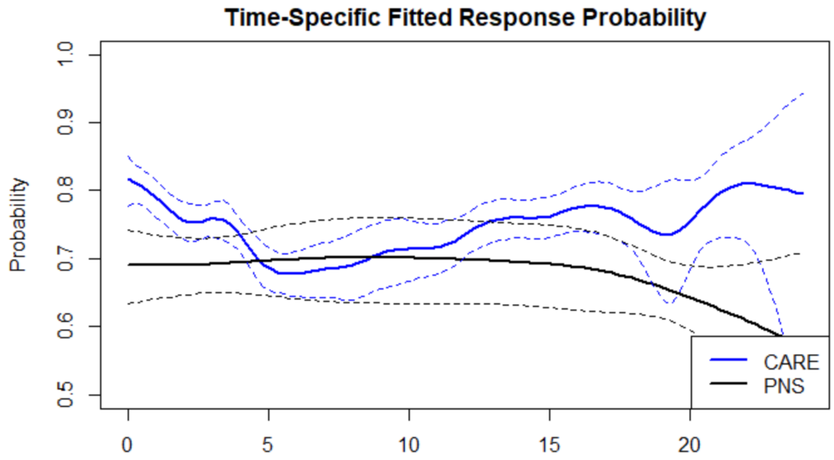

Supplementary Figure 7. Time-varying response rate across time from previous EMA prompt (0-24hrs) in the CARE and PNS datasets.
